# Cimetidine Attenuates Therapeutic Effect of Anti-PD-1 and Anti-PD-L1 and Modulates Tumor Microenvironment in Colon Cancer

**DOI:** 10.3390/biomedicines12030697

**Published:** 2024-03-21

**Authors:** Feng-Chi Kuo, Jerry Cheng-Yen Lai, Hui-Ru Shieh, Wan-Zu Liou, Ming-Jong Bair, Yu-Jen Chen

**Affiliations:** 1 Division of Nephrology, Department of Internal Medicine, Taitung MacKay Memorial Hospital, Taitung 950408, Taiwan; a5754@mmh.org.tw; 2 Department of Medical Research, Taitung MacKay Memorial Hospital, Taitung 950408, Taiwan; chengyen@gm.nttu.edu.tw; 3 Master Program in Biomedicine, College of Science and Engineering, National Taitung University, Taitung 950309, Taiwan; 4 Department of Medical Research, MacKay Memorial Hospital, New Taipei City 251020, Taiwan; ru123@mmh.org.tw; 5 Department of Radiology, Taitung MacKay Memorial Hospital, Taitung 950408, Taiwan; a1336@mmh.org.tw; 6 Division of Gastroenterology, Department of Internal Medicine, Taitung MacKay Memorial Hospital, Taitung 950408, Taiwan; 7 Department of Medicine, MacKay Medical College, New Taipei City 252005, Taiwan; 8 Department of Radiation Oncology, MacKay Memorial Hospital, Taipei 104217, Taiwan; 9 Department of Artificial Intelligence and Medical Application, MacKay Junior College of Medicine, Nursing and Management, Taipei 112021, Taiwan; 10 Department of Medical Research, China Medical University Hospital, Taichung 404332, Taiwan

**Keywords:** cimetidine, tumor microenvironment, immunomodulation

## Abstract

Histamine modulates immunity by binding to histamine receptor 2 (H2R). Cimetidine, an H2R antagonist that inhibits gastric acid secretion and treats gastrointestinal ulcers, interferes with histamine-mediated immunomodulation and may have anticancer activity. This study examined cimetidine’s effect on the anticancer effect of anti-PD-L1 in colon cancer. The MTT assay, colony formation assay, and DNA histograms assessed cell viability, clonogenicity, and cell cycle distribution, respectively. Flow cytometry measured H2R and PD-L1 expression and estimated specific immune cell lineages. For the in vivo study, tumor cells were subcutaneously implanted into the right flank of BALB/c mice. Cimetidine had no significant effect on CT26 cell viability, clonogenicity, or cell cycle distribution. It also did not affect H2R and PD-L1 expression levels in CT26 cells. In vivo, anti-PD-1 and anti-PD-L1 suppressed CT26 tumor growth, whereas cimetidine showed mild antitumor activity. In the combined experiment, cimetidine significantly attenuated anti-PD-1 and anti-PD-L1′ antitumor effects without major toxicity. In the tumor microenvironment, anti-PD-L1 increased CD3^+^ T, CD4^+^ T, and CD8^+^ T cells and M1 macrophages. Combined treatment with cimetidine reversed this. Cimetidine also reversed anti-PD-1 and anti-PD-L1′s decrease in circulating and tumor-associated neutrophils. Cimetidine attenuated anti-PD-L1′s antitumor effect and modulated the tumor microenvironment in colon cancer.

## 1. Introduction

Colon cancer is a gastrointestinal malignancy with increasing incidence worldwide [1]. Colorectal cancer (CRC) is categorized into two mutation profiles: tumors with mismatch repair deficiency and high-level microsatellite instability (dMMR-MSI-H CRC), and tumors with mismatch repair proficiency and low-level microsatellite instability (pMMR-MSI-L CRC) [2,3]. The dMMR-MSI-H CRC accounts for 15% of colorectal cases [3]. Current clinical trial results indicate that dMMR-MSI-H CRC is relatively sensitive to treatment with immune checkpoint inhibitors (ICIs), including PD-1/PD-L1 inhibitors, compared to pMMR-MSI-L CRC [4,5]. However, the response rates of ICIs for dMMR CRC, approximately 33% objective response, remain unsatisfactory [4,6,7]. Another issue to be addressed is improving the efficacy of ICIs in patients with pMMR CRC, which is a major CRC population. 

The tumor microenvironment (TME) consists of various cell populations, including cancerous and non-cancerous cells, immune cells, T and B cells, tumor-associated macrophages, neutrophils, dendritic cells (DCs), fibroblasts, endothelial cells, and adipocytes [8]. In the TME, the expression of immunosuppressive cells, such as regulatory T cells, M2 macrophages, or myeloid-derived suppressor cells, allows cancer cells to evade immune surveillance [9]. Other immune cells, such as natural killer (NK) cells, DCs, cytotoxic T cells, and M1 macrophages, play a role in suppressing tumor growth [10]. The TME also contains soluble mediators, extracellular matrix, and their interactions with cell lineages, making it complex and heterogeneous. 

Histamine exerts modulatory effects on both innate and adaptive immunity by binding to histamine receptor 2 (H2R). Cimetidine is a clinical H2R antagonist that inhibits gastric acid secretion for the treatment of gastrointestinal ulcers [11,12]. It has been demonstrated to interfere with histamine-mediated immunomodulation and may have anticancer activity against several types of malignancies, such as colorectal [13], gastric [14], kidney [15], and melanoma [16]. However, the role of H2R and its antagonists in modulating antitumor immunity remains controversial. In clinical practice, H2R antagonists, such as cimetidine, are commonly prescribed for the treatment and prevention of gastrointestinal ulcerative diseases, especially during stressful cancer therapy [17]. Cimetidine has been reported to exert anticancer and immunomodulatory effects by affecting both innate and adaptive immune responses [18,19]. Many clinical studies have revealed that cimetidine provides a survival benefit in patients with CRC after surgical resection. There are no reports on the effect of the combination of cimetidine and immunotherapy or its influence on the TME. Therefore, it is important to clarify whether H2R antagonists positively or negatively modulate the effects of immunotherapies, including ICIs.

In the present study, the combined effects of cimetidine and anti-PD-L1 on colon cancer growth and the TME were examined.

## 2. Materials and Methods

### 2.1. Cell Culture

Mouse colon adenocarcinoma CT26 cells derived from BALB/c mice were purchased from American Type Culture Collection (Manassas, VA, USA). Cells were cultured in RPMI-1640 medium (GIBCO, Grand Island, NY, USA), with 10% fetal bovine serum (Hyclone, Logan, UT, USA) and 200 mM of L-glutamine (Merck, Darmstadt, Germany) at 37 °C in a 5% CO_2_ incubator. Cells were maintained in an exponential growth pattern.

### 2.2. MTT Assay

An MTT assay was used to measure mitochondrial activity and estimate cell viability. Briefly, the cells were seeded in a 24-well plate and treated with cimetidine for 24 and 48 h. MTT was added into cell culture and incubated for 4 h at 37 °C. The medium was removed and DMSO was added for 30 min to dissolve the formazan crystals. The absorbance at 570/630 nm of each well was measured and determined by an ELISA reader. All experiments were performed in triplicate.

### 2.3. Clonogenic Assay

One hundred CT26 colorectal adenocarcinoma cells were cultured in 6-well plates for 24 h and then treated with cimetidine 0, 0.1, 1, 10, and 100 μM for 24 h. After treatment, cimetidine was removed, and the cells were incubated for 7 days. The cells were stained with 3% crystal violet to visualize colony formation. The surviving fraction was calculated as the number of inoculated cells multiplied by the plating efficiency.

### 2.4. Cell Cycle Analysis

Cells with different treatments were harvested, washed with phosphate buffer saline (PBS), and fixed in 70% (*v*/*v*) ethanol. Then the cells were washed, resuspended in cold PBS and incubated with 10 mg/mL RNase and 1 mg/mL propidium iodide at 37 °C for 30 min in the dark. The samples were analyzed by cell acquisition using a flow cytometer (BD FACSCalibur, Frankin Lakes, NJ, USA). The percentages of cancer cells distributed in the G0/G1, S, and G2/M phases were estimated using the ModFit LT software version 4.0. All experiments were performed in triplicate.

### 2.5. H2R, PD-1, and PD-L1 Expression Profile

After being treated with various concentrations of cimetidine for 24 h, cells were washed with PBS, harvested and stained with anti-H2R, anti-PD-1, and anti-PD-L1 antibodies for 30 min at 4 °C. The cells were stained with secondary antibody conjugated with fluorescein isothiocyanate for 30 min at 4 °C in the dark. Fluorescence was measured using a FACSCalibur flow cytometer (BD Biosciences, Frankin Lakes, NJ, USA). Data were collected and analyzed using the CellQuest Pro software version 6.1 (BD Biosciences).

### 2.6. Syngeneic Tumor Implantation Model

CT26 is an N-nitroso-N-methylurethane, undifferentiated Grade IV colon carcinoma cell line derived from BALB/c mice. CT26-bearing BALB/c mouse tumor animal models with stable microsatellite states have been widely used in research on CRC. In addition, researchers have used this model to evaluate the role of the TME in tumor development and dissemination. The experimental animals, BALB/c mice (five-week age), were obtained from the National Laboratory Animal Center (Taipei, Taiwan) and held in a specific pathogen-free environment of MacKay Memorial Hospital Laboratory Animal Center. The experiment protocols were performed in line with the rules and regulations, and authorized by the Experimental Animal Committee of MacKay Memorial Hospital (approval number: MMH-A-S-107-22). CT26 adenocarcinoma cells (4 × 10^6^) suspended in 50 μL PBS were implanted into the right hind leg of each mouse. While the tumors grew to reach a 0.5 cm diameter, mice were randomly allocated into six groups as follows: (1) control, (2) cimetidine (100 mg/kg five times a week via oral forced feeding), (3) intraperitoneal anti-mouse PD-1 (CD279) injection (200 μg per intraperitoneal injection every other day for a total of three times) (catalog number # BE0146, RMP1-14, BioXcell, Lebanon, NH, USA), (4) cimetidine in combination with anti-PD-1, (5) intraperitoneal anti-mouse PD-L1 (B7-H1) injection (150 μg per intraperitoneal injection every other day for a total of three times) (catalog number # BE0101, RMP1-14, BioXcell, Lebanon, NH, USA), and (6) cimetidine in combination with anti-PD-L1.

### 2.7. Evaluation of Tumor Volume and Toxicity

Each mouse’s body weight and the tumor size were measured every other day by a regular observe. The tumors were measured using electronic caliper to record the longest (a) and shortest (b) diameter of tumor and the volume was calculated using the formula 0.5ab^2^. For toxicity evaluation, blood samples were collected from the retro-orbital fossa using the micro hematocrit tube, and white blood cells were counted using an automatic Coulter counter (HEMAVET HV950; Drew Scientific, Inc., Dallas, TX, USA). The Fuji Dri Chem Slide (Fuji Dri Chem Slide, Fuji, Japan) was used to detect the plasma alanine aminotransferase and creatinine levels to represent liver and renal function, respectively, and the amounts were measured using a Fujifilm DryChem NX-500 analyzer (Fujifilm, Minato-ku, Tokyo, Japan).

### 2.8. Flow Cytometry Analysis of the Immune Cells

At the end of the experiment, the mice were euthanized with ketamine (100 mg/kg) and xylazine (10 mg/kg) for sacrifice. Tumors and spleens were harvested, minced into 2–4 mm small pieces. The specimens were digested with a solution containing collagenase A (1.5 mg/mL) and DNase I (0.4 mg/mL) at 37 °C for 30 min. The cells were filtered with the 70-μm cell strainer to separate and collect single-cell suspensions. Red blood cell (RBC) elimination was carried out using ammonium chloride–potassium solution (Invitrogen, Waltham, MA, USA) to lyse RBC. Before staining for cell surface markers, cells were suspended in the mouse BD Fc Block^TM^ reagent (1 μg/1 × 10^6^ cells; BD Bioscience, San Diego, CA, USA) at 37 °C for 1 h to prevent non-specific binding. Then, the cells were stained with antibodies conjugated to the different wavelength of fluorochromes for 20 min on ice in the dark. After washing with PBS, the cells were acquired using cytoFLEX 13-color cytometry (Beckman Coulter, Brea, CA, USA) to detect fluorescence and quantified using CytExpert analysis software version 2.3.0.84 (Beckman Coulter, Brea, CA, USA). Immune profiles were defined as: CD8^+^ T cells (CD3^+^/CD8^+^), CD4^+^ T cells (CD3^+^/CD4^+^), NKG2D^+^ T cells (CD3^+^/NKG2D^+^), macrophages (CD3^-^/CD11b^+^/Ly6C^+^/F4/80^+^/LY6G^-^), neutrophils (CD11b^+^/Ly6G^+^), NK cells (CD3^-^/CD11b^-^/NKG2D^+^/LY6G^-^), inflammatory monocytes (CD11b^+^/Ly6C^++^), M1 (CD45^+^/CD3^-^/LY6G^-^/F4/80^+^/MHCII^+^), M2 (CD45^+^/CD3^-^/LY6G^-^/F4/80^+^/CD206^+^), DCs (CD45^+^/CD3^-^/LY6G^-^/F4/80^+^/CD11c^+^), and regulatory T cells (CD45^+^/CD3^+^/LY6G^-^/CD4^+^/FoxP3^+^).

### 2.9. Statistical Analysis

Data are expressed as mean (standard error of the mean). One-way analysis of variance (ANOVA) and Fisher’s least significant difference (LSD) test were used for pairwise comparisons of means. A repeated-measures analysis of variance and a generalized estimating equation (GEE) were used to adjust for the correlation of repeated measurements. All data transformations and statistical analyses were performed using IBM SPSS statistical software (version 25) (IBM Corp., Armonk, NY, USA). All statistical significances between groups are indicated by * *p* < 0.05, ** *p* < 0.01, and *** *p* < 0.001.

## 3. Results

### 3.1. Effect of Cimetidine on Cell Viability, Clonogenicity, and Cell Cycle Distribution

**Figure 1:**
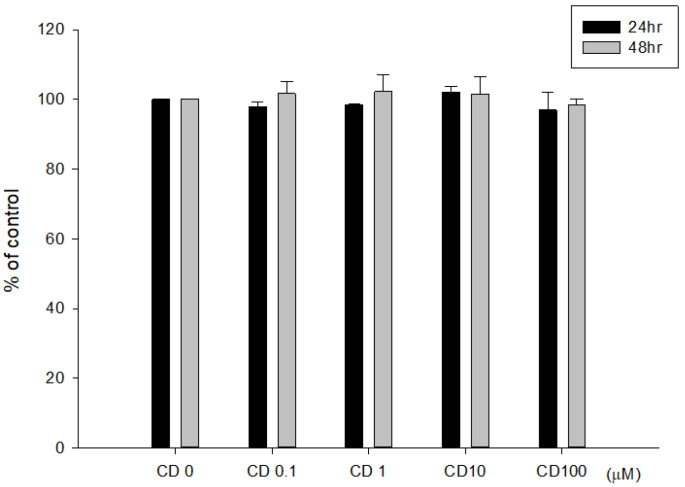
Cell viability of colon adenocarcinoma CT26 cells after cimetidine treatment. CT26 cells were treated with cimetidine (CD) 0 to 100 μM for 24 and 48 h; cell viability was detected by MTT reduction assay. Data from three separate experiments are expressed as mean ± standard error of the mean.

**Figure 2:**
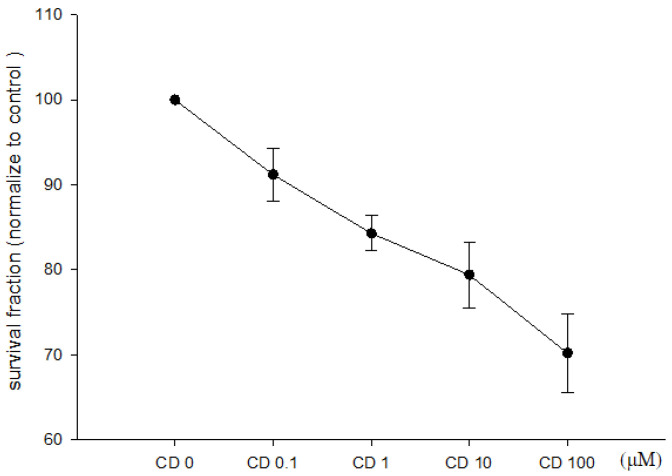
Clonogenic assay of colon adenocarcinoma CT26 cells after cimetidine treatment. CT26 cells were seeded on the plate and treated with cimetidine (CD) 0 to 100 μM for 24 h, then the cimetidine was washed. After one week, cells were stained with crystal violet to visualize colony formation and numbers were counted. Data from three separate experiments are expressed as mean ± standard error of the mean.

**Figure 3:**
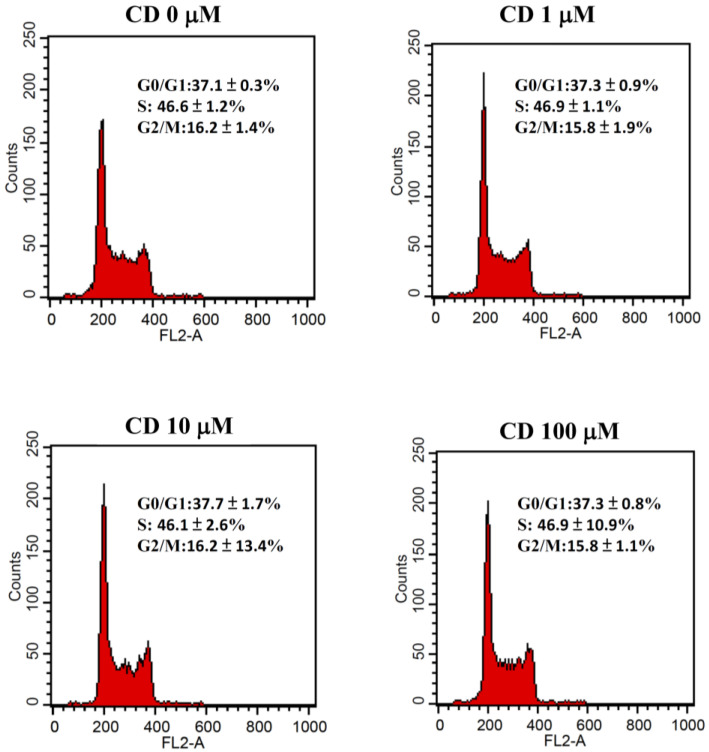
Cell cycle distribution of colon adenocarcinoma CT26 cells after cimetidine treatment. CT26 cells were seeded on the plate and treated with cimetidine (CD) 0 to 100 μM for 24 h, then cells were harvested, fixed, and stained with propidium iodide. Each phase of cell cycle was acquired by flow cytometry and analyzed by ModFit LT software version 4.0. Data from three separate experiments are expressed as mean ± standard error of the mean.

The direct effects of cimetidine on the viability, clonogenicity, and cell cycle distribution of CT26 cells were examined to determine whether cimetidine possesses cytotoxic activity against CT26 cells. Cimetidine had no significant effect on cell viability, as evaluated by the MTT assay (Figure 1). The clonogenicity of CT26 cells was significantly affected by cimetidine (Figure 2). The DNA histogram for cell cycle analysis of CT26 cells showed no significant alterations following cimetidine treatment (Figure 3).

### 3.2. Effect of Cimetidine on Expression of Surface H2R and PD-L1

**Figure 4:**
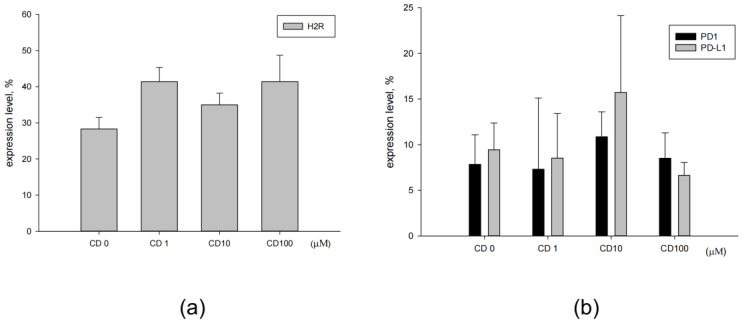
(**a**,**b**) Expression levels of H2R, PD-1, and PD-L1 on colon adenocarcinoma CT26 cells after cimetidine treatment. CT26 cells were treated with cimetidine (CD) 0 to 100 μM for 24 h, then cells were harvested, fixed, and stained with anti-H2R (**a**), anti-PD-1, and anti-PD-L1 (**b**) antibodies. Fluorescence intensity was acquired by flow cytometry and the fluorescence intensity was measured by CellQuest Pro software version 6.1. Data from three separate experiments are expressed as mean ± standard error of the mean.

The expression of H2R on the surface of CT26 cells treated with 0, 1, 10, and 100 μM cimetidine was 28.3 ± 3.2%, 41.4 ± 3.9%, 35.8 ± 3.2%, and 41.4 ± 7.3%, respectively (Figure 4a). The expression of PD-L1 on CT26 cells was 9.4 ± 2.9%, which was not affected by cimetidine with 8.5 ± 4.9%, 15.7 ± 8.4%, and 6.6 ± 1.4% for 1, 10, and 100 μM. The PD-1 expressed on CT26 cells was 7.8 ± 3.2%, 7.3 ± 7.8%, 10.9 ± 2.7%, and 8.5 ± 2.8% for 0, 1, 10, and 100 μM of cimetidine, respectively (Figure 4b).

### 3.3. Effect of Cimetidine and Anti-PD-1/Anti-PD-L1 on CT26 Tumor Growth and Toxicity

**Figure 5:**
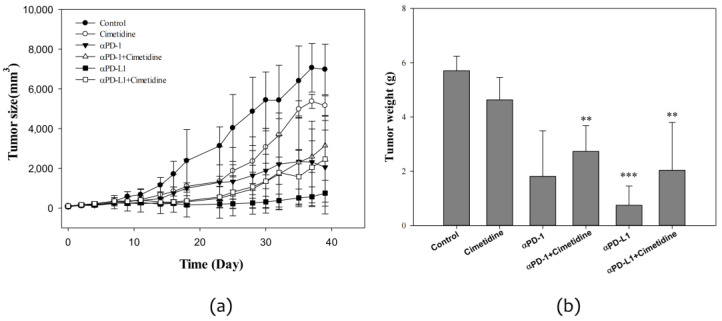
Therapeutic effect in CT26 syngeneic animal model after cimetidine, anti-PD-1, anti-PD-L1, combination of cimetidine and anti-PD-1, and cimetidine and anti-PD-L1 treatment. BALB/c mice were implanted with CT26 colon adenocarcinoma cells for tumor growth. One week later, mice were administered with cimetidine (100 mg/kg), anti-PD-1 antibody (200 μg), anti-PD-L1 antibody (150 μg), or combination of cimetidine and anti-PD1, and cimetidine and anti-PD-L1 antibody. Tumor volume was recorded for a month using an electronic caliper (**a**). At the end, mice were sacrificed and the tumors were harvested and weighed (**b**). Data from four mice of each group are expressed as mean ± standard error of the mean. Significant differences between control group and treated-groups are indicated by ** *p* < 0.01, and *** *p* < 0.001.

**Figure 6:**
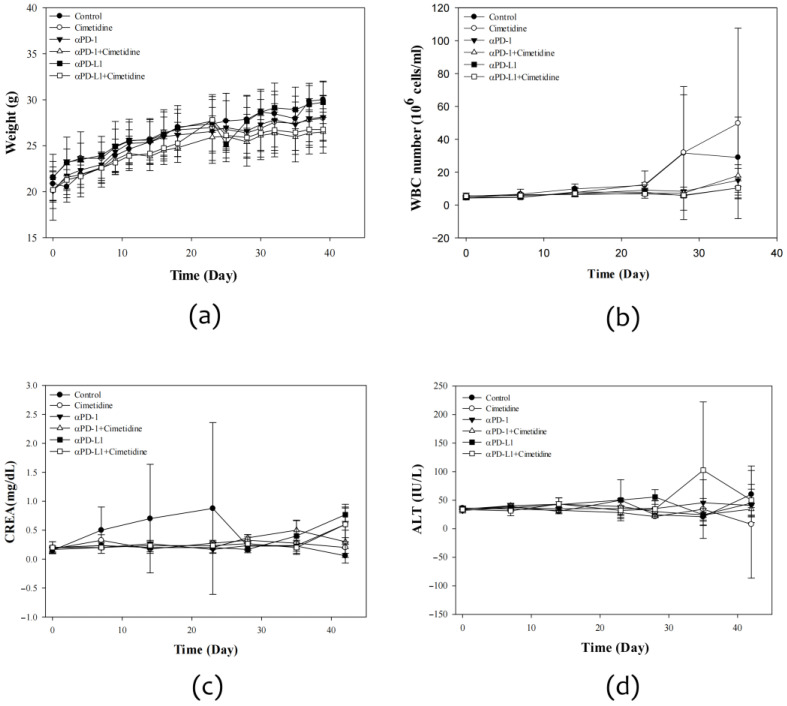
Toxicity effect in CT26 syngeneic animal model after cimetidine, anti-PD-1, anti-PD-L1, combination of cimetidine and anti-PD-1, and cimetidine and anti-PD-L1 treatment. BALB/c mice were implanted with CT26 colon adenocarcinoma cells for tumor growth. One week later, mice were administered with cimetidine (100 mg/kg), anti-PD-1 antibody (200 μg), anti-PD-L1 antibody (150 μg), or combination of cimetidine and anti-PD1, and cimetidine and anti-PD-L1 antibody. Biological toxicities were evaluated by body weight (**a**), white blood cell (WBC) counts (**b**), creatinine (CREA) for renal function (**c**), and alanine transferase (ALT) for liver function (**d**). Data from four mice of each group are expressed as mean ± standard error of the mean.

In vivo, anti-PD-L1 profoundly suppressed CT26 tumor growth, whereas cimetidine showed mild antitumor activity. In a combined experiment, cimetidine significantly attenuated the antitumor effect of anti-PD-L1. The tumor weight at the 39th post-implantation day was 5.7 ± 0.5, 4.6 ± 0.8, 1.8 ± 1.7, 2.7 ± 1.0, 0.8 ± 0.7, and 2.0 ± 1.8 g for control, cimetidine, anti-PD-1, anti-PD-1 plus cimetidine, anti-PD-L1, and anti-PD-L1 plus cimetidine, respectively (Figure 5). With regard to major toxicity, no significant alterations were observed after adding cimetidine to anti-PD-1 or anti-PD-L1 in terms of body weight, white blood cell count, plasma alanine aminotransferase levels, or plasma creatinine levels (Figure 6).

### 3.4. Effect of Cimetidine and Anti-PD-1/Anti-PD-L1 on Immune Cells Expression in Spleen and Tumor Microenvironment 

**Figure 7:**
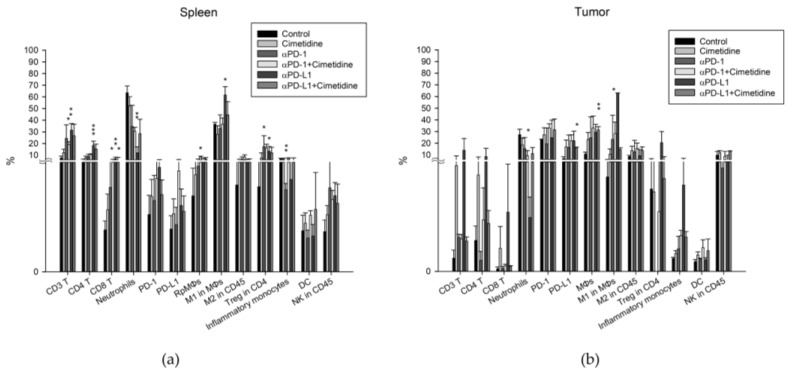
Expression profiles of different types of immune cells in CT26 syngeneic animal model after cimetidine, anti-PD-1, anti-PD-L1, combination of cimetidine and anti-PD-1, or cimetidine and anti-PD-L1 treatment. Mice were sacrificed and tumor and spleen specimens were harvested, minced, and dissociated with single cells for flow cytometry to acquire immune cells. Different lineages of immune cells in spleen (**a**) and tumor (**b**) expression were quantified on day 39 after CT26 cells were implanted in BALB/c mice. Data from four mice of each group are expressed as mean ± standard error of the mean. Significant differences between control group and treated-groups are indicated by * *p* < 0.05, ** *p* < 0.01, and *** *p* < 0.001.

For both circulating and TME neutrophils, anti-PD-L1 decreased the amounts, and cimetidine reversed this effect (Figure 7a,b). Anti-PD-1 and anti-PD-L1 alone slightly increased the number of regulatory T cells, but did not show alterations after combination therapy. This suggests that the increase in regulatory T cells may not play an important role in mono- or combination therapy (Figure 7a). In the TME, anti-PD-L1 increased the number of CD3^+^ T, CD4^+^ T, and CD8^+^ T cells and M1 macrophages, which was reversed by combined treatment with cimetidine.

## 4. Discussion

The H2 receptor antagonist cimetidine reduces the antitumor activity of anti-PD-L1 in a syngeneic CT26 colon cancer experimental model. This attenuation effect was accompanied by modulation of the TME.

Histidine decarboxylation by histidine decarboxylase results in the formation of histamines. Histamine is a major mediator that binds to surface receptors on cells and induces allergies and inflammation. In allergic reactions, secreted immunoglobulin E binds to its receptors on mast cells, and basophils initiate cell activation and histamine production [20]. Histamine release causes allergic effects in various systems, such as the gastrointestinal, respiratory, and cardiovascular systems. Histamine is also a neurotransmitter in the central nervous system that influences food intake [21] and sleep mechanism [22]. Tryptophan catabolism involves two major enzymatic pathways resulting in the production of 5-hydroxytryptamin and kynurenine. In the kynurenine pathway, tryptophan is catabolized to kynurenine by tryptophan 2,3-dioxygenase in the liver and by indoleamine 2,3-dioxygenase in other tissues. Kynurenine can be further metabolized into downstream products collectively referred to as kynurenine metabolites, including 3-hydroxykynurenine, xanthurenic acid, anthranilic acid, 3-hydroxyanthranilic acid, and picolinic acid. Kynurenine metabolites play an important role in energy metabolism and immune modulation. The kynurenine pathway can be activated to generate NAD+ as an energy source for activated immune cells. In contrast, kynurenine and 3-hydroxyanthranilic acid may be involved in the apoptosis of Th1 and NK cells, leading to immunosuppression [23]. There is no direct link between the histidine and kynurenine metabolism pathways. However, these two pathways may have coexisting immunomodulatory effects, such as allergic reactions, on immune cells.

The antitumor effect of cimetidine has been suggested to involve various mechanisms underlying its beneficial effects in patients with cancer. In addition to its immunomodulatory effects, cimetidine exerts antitumor activity by inhibiting cancer cell proliferation by blocking histamine receptors [24], affecting histamine metabolism [25], blocking the expression of E-selectin to inhibit cancer cell adhesion and prevent metastasis [26], and inhibiting angiogenesis [27,28]. In our results, cimetidine had mild antitumor activity, but significantly attenuated the antitumor effect of anti-PD-L1 without major toxicity. The antitumor activity of cimetidine may occur through mechanisms other than immunomodulation. The mechanisms by which cimetidine inhibits tumor growth require further investigation. Clinical data have revealed that cimetidine is a safe drug with low toxicity and side effects [29]. The dose of cimetidine could exceed 800 mg/day for two months to treat peptic ulcers. This indicated that the cimetidine dose administered in our study was safe without major toxicity.

Cimetidine has been shown to exert anticancer effects in vitro and in vivo. Immunomodulation is a possible mechanism through which cimetidine inhibits tumor growth. Cimetidine can reverse the histamine-associated immunosuppressive effect and regulate immune cell activities, such as increasing DC antigen-presenting activity [30], augmenting NK cell activity [31], enhancing the tumor-infiltrating lymphocyte response [13,18,32], decreasing myeloid-derived suppressor cell activity [33], or activating NK cells [34] in the TME. Our TME results demonstrated that cimetidine increased the CD3^+^, CD4^+^, and CD8^+^ T cell populations. This indicates that cimetidine may augment T cell population reservoirs to enhance immune activity. However, DCs and NK cells did not show any alterations in our study.

The expression of various cytokines plays a crucial role in the immunomodulatory effects of cimetidine. Takahashi et al. have validated that a subcutaneously administered extremely low concentration of cimetidine (0.12 mg/kg/day) suppressed CT26 tumor growth and detected various cytokines’ mRNA expression. They found that restoration of decreased cytokines, such as lymphotoxin-β, TNF-α, IFN-γ, IL-10, and IL-15, by cimetidine in tumors is associated with antitumor effect [35]. The levels of proinflammatory cytokines were significantly decreased by cimetidine in ICR mice [36]. Clinically, the differential ability of cells is not affected in normal individuals or patients with CRC [30]. However, cimetidine elevates the antigen-presenting capacity of DCs in patients with CRC and increases this capacity in patients with advanced CRC. These may be the mechanisms by which cimetidine modulates the TME. Cytokine expression or antigen-presenting capacity in the TME should be further investigated and elucidated.

The CT26 cell line is characterized as a pMMR cell line [37]. In this study, we found that cimetidine in combination with anti-PD-L1 to treat CT26 syngeneic mice demonstrated an attenuated tumor control. In the TME, cimetidine reversed the increase in the numbers of CD3^+^, CD4^+^, and CD8^+^ T cells and M1 macrophages induced by anti-PD-L1. These cell lineages are regarded as possessing antitumor capacity. For both circulating and tumor-associated neutrophils, the proposed cell ontogeny with pro-tumoral features was decreased by anti-PD-L1, and cimetidine reversed this effect. Collectively, our findings provide evidence that cimetidine may act as an immunomodulatory agent to reduce the sensitivity of pMMR CRC tumors to anti-PD-L1 therapy. Whether the negative impact of cimetidine in mice can translate into human practice should be further validated in clinical trials.

In this study, the CT26 cells expressed 28.3 ± 3.2% of H2R and 9.4 ± 2.9% of PD-L1, which were not affected by cimetidine treatment in vitro. The surface expression of PD-L1 on other colon cancer cells, such as SW480 and HCT116, is 5.41 ± 0.06% and 2.77 ± 0.06%, respectively [38]. In our study, the surface expression of PD-L1 in CT26 cells was higher than that in the other colon cancer cell lines. In the clinical application of ICI therapy, the expression of PD-L1 in tumors or tumor-infiltrating leukocytes is regarded as a companion test for anti-PD-L1/anti-PD-1. If cimetidine acts through H2R in this scenario, examination of H2R expression in tumors or tumor-infiltrating leukocytes might be feasible. Whether H2R could be a unique target for the modulation of the TME and antitumor immune response should be considered in future investigations. 

Immunotherapy has been implicated in the treatment of many cancer types since it was first approved by the Food and Drug Administration of the United States for melanoma in 2011. To date, the response rate to immunotherapy is only 20–40% in patients [39]. The development of a new combination therapy with immunotherapy is crucial for cancer immunotherapy. The dose of cimetidine used in our study was relatively low, and its contradictory effects on immune checkpoint blockade are important in clinical practice. Given that cimetidine is commonly used for both the treatment and prevention of peptic ulcers, the results of this study may provide a clinical warning against the easily neglected prescription of PD-1/PD-L1 inhibitors.

## 5. Conclusions

In conclusion, cimetidine, an H2R blocker, may attenuate the anti-PD-1 and anti-PD-L1 effects and modulate the circulating and TMEs in colon cancer. Previous studies have revealed that cimetidine possesses anticancer ability in multiple preclinical cancer types and provides survival benefits in clinical data. A limitation of this study is the use of CT26-bearing BALB/c mice for CRC. Different animal models, such as tumor-bearing C57BL/6 mice with various cancer types, can be used to evaluate the effects of cimetidine combined with immunotherapy. Therefore, in future studies, it will be possible to develop combination therapies with other regimens to treat cancer.

## Data Availability

Data are contained within the article.
